# Myocardial Protection Efficacy of Custodiol, Del Nido, and Cold Intermittent Blood Cardioplegia in Arterial Switch Operation

**DOI:** 10.1093/icvts/ivaf215

**Published:** 2025-09-24

**Authors:** Mustafa Kemal Avşar, Yasin Güzel, Barış Kırat, İbrahim Özgür Önsel, Cenap Zeybek, Deniz Yorgancılar, İlker Kemal Yücel

**Affiliations:** Department of Cardiovascular Surgery, Çukurova University Faculty of Medicine, Adana 01330, Turkey; Department of Cardiovascular Surgery, Çukurova University Faculty of Medicine, Adana 01330, Turkey; Department of Anesthesiology and Reanimation, Medicana International Istanbul Hospital, Istanbul 34520, Turkey; Department of Anesthesiology and Reanimation, Medicana International Istanbul Hospital, Istanbul 34520, Turkey; Department of Pediatric Cardiology, Medipol Mega University Hospital, Istanbul 34214, Turkey; Department of Thoracic Surgery, Medicana International Istanbul Hospital, Istanbul 34520, Turkey; Department of Pediatric Cardiology, Istanbul University - Cerrahpasa Medical Faculty, Istanbul 34098, Turkey

**Keywords:** arterial switch operation, cardioplegia, Custodiol, Del Nido, cold blood cardioplegia, neonatal cardiac surgery, myocardial protection, transposition of the great arteries

## Abstract

**Objectıves:**

The arterial switch operation (ASO) is the standard treatment for transposition of the great arteries (TGA), requiring robust myocardial protection due to the neonatal myocardium’s vulnerability to ischaemia. This study compares the myocardial protective efficacy of Custodiol, Del Nido, and cold intermittent blood cardioplegia in neonates undergoing ASO.

**Methods:**

We retrospectively analysed 133 neonates with TGA undergoing ASO (2013-2024) at 4 Turkish centres, grouped by cardioplegia: cold blood (*n* = 47), Custodiol (*n* = 44), or Del Nido (*n* = 42). Outcomes included aortic cross-clamp and cardiopulmonary bypass times, troponin I, CK-MB, inotropic support, and ventilation duration.

**Results:**

Custodiol and Del Nido had shorter cross-clamp (70.4 (8.5) vs 68.7 (7.9) vs 78.2 (9.1) minutes, *P* < .001) and bypass times (*P* = .004), lower troponin I (4.2 (1.3) vs 4.0 (1.5) vs 6.8 (1.9) ng/mL, *P* < .001), reduced inotropic needs (*P* < .001), and shorter ventilation/intensive care unit stays (*P* ≤ .010). Mortality was similar (*P* = .47).

**Conclusıons:**

Custodiol and Del Nido cardioplegia strategies provided favourable outcomes compared to cold blood cardioplegia in neonates undergoing ASO, with implications for optimizing myocardial protection protocols in this population.

## Graphical Abstract

**Figure:**
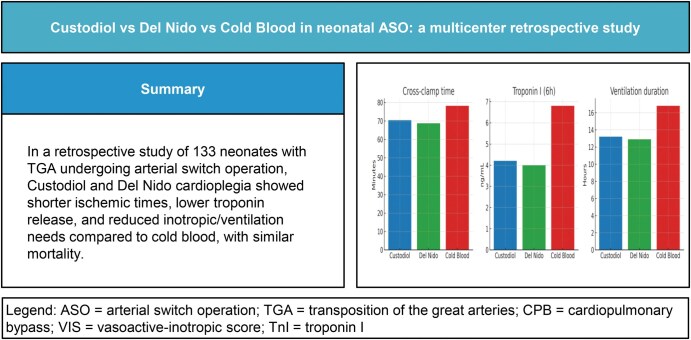

## INTRODUCTION

Transposition of the great arteries (TGA) is a critical congenital heart defect requiring arterial switch operation (ASO).^1^ Performed within the first 2 weeks, ASO demands robust myocardial protection due to the neonatal myocardium’s susceptibility to ischaemia from immature metabolic pathways.^2^^,^^3^ Cardioplegia solutions minimize ischaemia-reperfusion injury during cardiopulmonary bypass (CPB), but the optimal strategy for neonates remains debated.^4^^,^^5^

Cold blood cardioplegia has long been a reliable standard, despite its drawbacks of frequent dosing and prolonged operative times.^6^ In contrast, Custodiol and Del Nido solutions, designed for single-dose administration and prolonged myocardial protection, have emerged as promising alternatives in both paediatric and adult cardiac surgery.^6–8^ However, their efficacy in neonates is controversial.^8–13^ This study compares Custodiol, Del Nido, and cold blood cardioplegia in 133 neonates undergoing ASO, assessing intraoperative and postoperative outcomes to guide myocardial protection strategies.

## METHODS

### Study design and centres

**Figure 1.**
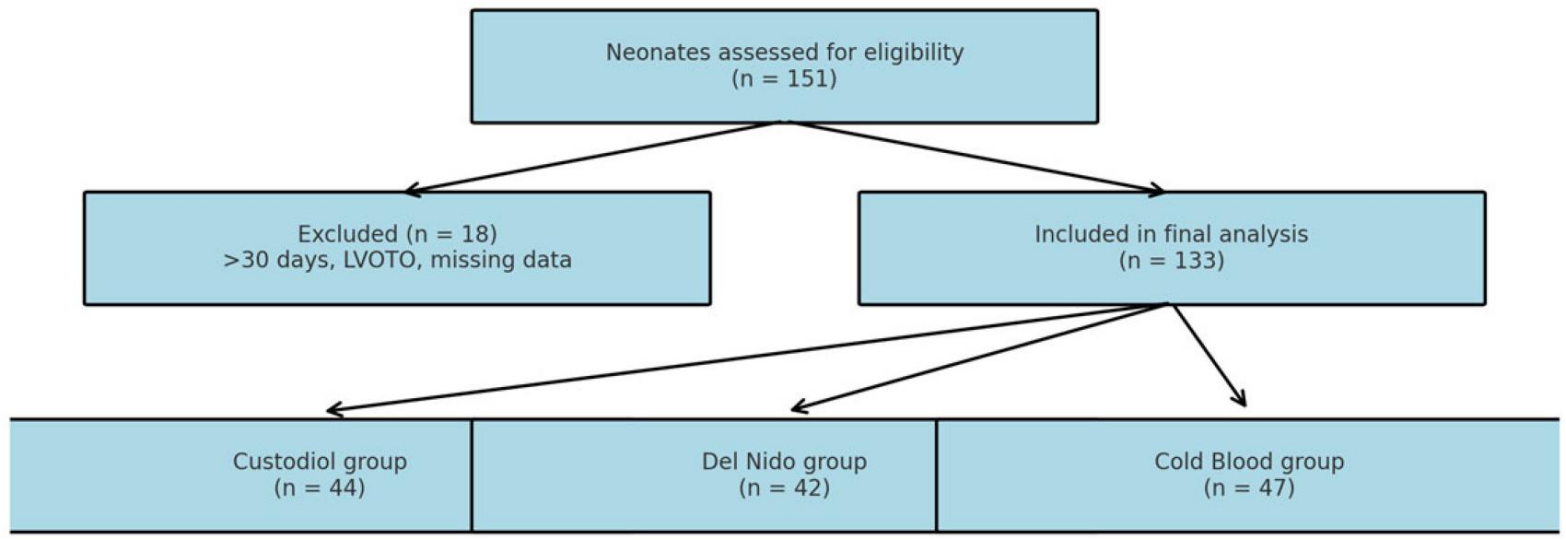
Flow Diagram Illustrating the Patient Selection Process for the Comparative Analysis of Cardioplegia Methods in 133 Neonates Undergoing Arterial Switch Operation. Exclusion criteria included age >30 days, missing data, and presence of left ventricular outflow tract obstruction (LVOTO)

This retrospective cohort study compared Custodiol, Del Nido, and cold blood cardioplegia in 133 neonates undergoing ASO for TGA. A single congenital cardiac surgeon performed all operations (2013-2024) across 4 centres. Cardioplegia strategy evolved over time: cold blood (2013-2016), Del Nido (2016-2020), and Custodiol (2020-2024). Patient selection is shown in **Figure 1**. Patients were grouped by cardioplegia:

- Cold blood (*n* = 47)
- Custodiol (*n* = 44)
- Del Nido (*n* = 42)

### Inclusion criteria

Neonates were included if they met the following criteria:

1. Confirmed diagnosis of d-TGA, with or without associated intracardiac anomalies (eg, ventricular septal defect [VSD], atrial septal defect [ASD], patent ductus arteriosus [PDA]).
2. Underwent primary ASO performed under CPB with cardioplegic arrest.
3. Surgery timing based on anatomical subtype:
  - Simple d-TGA with intact ventricular septum (IVS): surgery within the first 15 days of life
  - d-TGA with VSD: surgery within the first 30 days of life
4. Complete intraoperative and early postoperative data available, including aortic cross-clamp time, troponin I, CK-MB, inotropic support, and ventilation duration.

### Exclusion criteria

- Underwent procedures other than ASO.
- Received staged palliation instead of primary ASO.
- Were older than 30 days at the time of surgery.
- Had major extracardiac anomalies or known genetic syndromes that could affect surgical outcomes.
- Had incomplete intraoperative or early postoperative data.
- Had TGA with left ventricular outflow tract obstruction (LVOTO).

### Cardioplegia protocols

#### Cold intermittent blood cardioplegia

A cardioplegic solution composed of oxygenated blood and crystalloid in a 4:1 ratio, containing potassium chloride (25 mmol/L), sodium bicarbonate (10 mmol/L), and mannitol (30 mmol/L). It was administered antegrade at 4-8 °C and repeated every 15 minutes throughout the cross-clamp period.

#### Custodiol (HTK solution)

A crystalloid-based, intracellular-type cardioplegia composed of histidine (198 mmol/L), potassium chloride (9 mmol/L), magnesium chloride (4 mmol/L), tryptophan (2 mmol/L), α-ketoglutarate (1 mmol/L), and mannitol (30 mmol/l). It was administered antegrade at 4-8 °C as a single dose of 50 ml/kg over 3-5 minutes, providing up to 180 minutes of myocardial protection.

#### Del Nido cardioplegia

A 4:1 blood-to-crystalloid mixture based on Plasma-Lyte A, containing lidocaine (1 mmol/L), potassium chloride (13 mmol/L), magnesium sulphate (16 mmol/L), mannitol (30 mmol/L), and sodium bicarbonate (13 mmol/L). It was delivered antegrade at 4 °C over 3-5 minutes, offering myocardial protection for 60-90 minutes.

### Surgical technique and patient selection

All procedures were performed via median sternotomy under standardized general anaesthesia.

Cardiopulmonary bypass was established using aortic and bicaval cannulation under mild hypothermia (30-32 °C). CPB flow rate was maintained at 150-170 ml/kg/minutes, with a target mean arterial pressure of 30-40 mmHg. Haematocrit was maintained at 28%-32%; MUF was applied post-CPB.

Myocardial protection was achieved via antegrade infusion of 1 of the 3 cardioplegia solutions immediately after aortic cross-clamping: cardioplegia administration was performed as described in Section “Cardioplegia protocols”.

Arterial switch operation was performed using standard techniques, including Lecompte manoeuvre and coronary reimplantation (trap-door technique). Associated intracardiac anomalies (eg, VSD, aortic coarctation) were repaired concurrently. In cases with aortic arch obstruction, an extended end-to-end anastomosis was performed under circulatory arrest or low-flow CPB as needed. Coronary artery transfer used the trap-door technique, except in 6 patients (4.5%) with intramural left main coronary artery (LMCA), where unroofing and patch augmentation (typically autologous pericardium) were applied. Sixteen patients (12%) had single coronary ostium or atypical coronary patterns (eg, retroaortic circumflex, anterior looping), requiring individualized implantation strategies. No intraoperative or early postoperative coronary-related complications were observed.

The mean anaesthetic induction time (from induction to skin incision) was 35 ± 8 minutes, consistent across groups. No significant changes were made to operative or anaesthetic protocols during the study period. Patients with complex anatomical anomalies, such as LVOTO, were excluded to ensure homogeneity in myocardial protection assessment.

Anatomical characteristics included:

- VSD in 69 patients (51.8%).
- Aortic coarctation in 7 patients (5.3%).
- Coronary anomalies: intramural LMCA in 6 patients (4.5%), single coronary artery origin in 11 patients (8.3%), retroaortic circumflex in 5 patients (3.8%).
- Great artery orientation: anterior-posterior in 116 patients (87.2%), side-by-side in 17 patients (12.8%).

### Echocardiographic assessment

Transthoracic echocardiography (TTE) was performed preoperatively, early postoperatively (within 1 hour), and at hospital discharge using standard imaging protocols. Left ventricular function was assessed qualitatively (normal, mild, moderate, or severe dysfunction) and quantitatively via left ventricular ejection fraction (LVEF) using the Simpson biplane method when feasible. Regional wall motion abnormalities (RWMA) were evaluated in apical 4-chamber, parasternal long-axis, and parasternal short-axis views. Most patients showed prompt ventricular function recovery post-reperfusion, with moderate-to-severe RWMA incidence below 5% at discharge. Three patients developed low cardiac output syndrome (LCOS) requiring inotropic and ventilatory support in the intensive care unit.

### Postoperative outcome measures

Postoperative outcomes included cross-clamp and CPB times, troponin I, CK-MB, vasoactive-inotropic score (VIS), ventilation duration, and intensive care unit (ICU) stay. VIS was calculated within 24 hours postoperation based on inotropic doses. VIS was calculated using the following formula: dopamine (μg/kg/minute) + dobutamine (μg/kg/minute) + 100 × epinephrine (μg/kg/minute) + 10 × milrinone (μg/kg/minute) + 10 000 × vasopressin (U/kg/minute) + 100 × norepinephrine (μg/kg/minute). Peak VIS within the first 24 hours postoperatively was recorded. All centres followed similar inotropic protocols based on national paediatric cardiac ICU recommendations.

Troponin I and CK-MB were measured serially postoperation to assess myocardial injury. Low cardiac output syndrome was defined as requiring inotropic and ventilatory support due to inadequate cardiac output, confirmed by echocardiographic ventricular dysfunction.

### Postoperative management and sternal closure strategy

Postoperative care protocols were broadly similar across all 4 centres. Early extubation and fast-track ICU discharge were encouraged when haemodynamic stability permitted. Minor differences in timing existed due to institutional practices, but the overall management approach was consistent.

All patients underwent attempted primary chest closure in the operating room. However, delayed sternal closure was performed in cases of haemodynamic instability or excessive bleeding. A total of 30 patients (22.6%) underwent delayed chest closure, including 9 in the Custodiol group (20.5%), 7 in the Del Nido group (16.7%), and 14 in the Cold Blood group (29.8%).

### Statistical analysis

Analyses used SPSS 26.0. Continuous variables (mean (SD) or median [IQR]) were compared via ANOVA or Kruskal-Wallis tests; categorical variables (*n*, %) via chi-square or Fisher’s exact tests (*P* < .05 significant). Normality was assessed using Shapiro-Wilk test. Normal variables used 1-way ANOVA with Bonferroni correction; non-normal variables used Kruskal-Wallis with Dunn’s *post hoc* test. AUC for hs-cTnI was calculated to assess myocardial injury and compared using ANOVA or Kruskal-Wallis based on distribution. Complete data for all patients’ primary and secondary outcomes allowed complete-case analysis without imputation.

## RESULTS

### Baseline characteristics

Of the 133 neonates undergoing ASO for TGA, 44 received Custodiol, 42 Del Nido, and 47 cold blood cardioplegia. Baseline characteristics (**Table 1**) differed in age, birth weight, preoperative oxygen saturation, and inotropic support (*P* < .05), reflecting clinical heterogeneity.

**Table 1.** Baseline Characteristics of the Study Population

Variable	Custodiol (*n* = 44)	Del Nido (*n* = 42)	Cold blood (*n* = 47)	*P*-value
Age at surgery (days)	10.3 (2.2)	13.9 ± 2.3	17.4 (1.5)	<.0001
Birth weight (kg)	2.7 (0.2)	3.2 ± 0.2	3.3 (0.3)	<.0001
Preoperative oxygen saturation (%)	58.0 (5.6)	73.7 ± 5.3	84.3 (5.4)	<.0001
Preoperative inotropic support, *n* (%)	33 (75%)	27 (64%)	22 (47%)	.0152
Presence of VSD, *n* (%)	26 (59%)	18 (43%)	25 (53%)	.3347
Presence of ASD or PFO, *n* (%)	32 (73%)	26 (62%)	33 (70%)	.5035
Balloon atrial septostomy, *n* (%)	15 (34%)	11 (26%)	8 (17%)	.1704
Preoperative lactate (mmol/L)	4.8 (2.0)	4.6 (1.3)	3.2 (1.1)	<.0001

Data are presented as mean (SD) or *n* (%). *P*-values were derived from 1-way ANOVA for continuous variables and chi-square or Fisher’s exact test for categorical variables.

Surgical and anatomical complexity variables are summarized in **Table 2**.

**Table 2.** Surgical and Anatomical Complexity by the Cardioplegia Group

Variable	Custodiol (*n* = 44)	Del Nido (*n* = 42)	Cold blood (*n* = 47)	*P*-value
Presence of VSD (%)	59.0	43.0	53.0	.33
Aortic coarctation (%)	9.0	2.0	4.0	.18
Intramural LMCA (%)	4.5	4.8	4.2	.96
Single coronary origin (%)	11.4	7.1	6.3	.59
Retroaortic circumflex (%)	4.5	4.8	2.1	.60
Aortic root orientation: AP (%)	86.0	86.0	89.0	.87
Aortic root orientation: side-by-side (%)	14.0	14.0	11.0	.87
Single coronary ostium (%)	11.4	7.1	6.3	.59
Intramural coronary course (%)	4.5	4.8	4.2	.96
Aortic arch anomaly (%)	9.0	2.0	4.0	.23

### Intraoperative and early postoperative outcomes

Custodiol and Del Nido groups showed shorter cross-clamp (70.4 (8.5) and 68.7 (7.9) vs 78.2 (9.1) minutes, *P* < .001) and CPB times (102.3 (10.5) and 100.8 (9.8) vs 108.6 (11.2) minutes, *P* = .004) than cold blood (**Table 2**). Custodiol had the highest cardioplegia volume (500 (50) ml), followed by Del Nido (400 (40) ml) and cold blood (320 (60) ml, *P* < .001). Repeat dosing occurred in 100% of cold blood, 8% of Del Nido, and 0% of Custodiol cases (*P* < .001). Custodiol and Del Nido had faster sinus rhythm recovery (*P* = .002) and fewer defibrillations (*P* = .034). Troponin I and CK-MB, measured at 6-72 hours across centres, used standardized assays. Custodiol and Del Nido had lower postoperative troponin I (4.2 (1.3) and 4.0 (1.5) vs 6.8 (1.9) ng/mL, *P* < .001), CK-MB, lactate, and inotropic scores (*P* < .001), plus shorter ventilation (13.2 (3.9) and 12.9 (3.6) vs 16.8 (4.5) hours, *P* = .001) and ICU stays (3.5 (1.2) and 3.4 (1.0) vs 4.2 (1.3) days, *P* = .010). LCOS was less frequent in Custodiol (4%) and Del Nido (2%) vs cold blood (14%, *P* = 0.048). Thirty-day mortality was similar (2.4%-6.4%, *P* = .47). Delayed sternal closure occurred in 30 patients (22.6%) due to low cardiac output or bleeding: Custodiol (*n* = 9), Del Nido (*n* = 7), cold blood (*n* = 14) (**Table 3**).

**Table 3.** Intraoperative and Early Postoperative Outcomes by the Cardioplegia Group

Parameter	Cold blood (*n* = 47)	Custodiol (*n* = 44)	Del Nido (*n* = 42)	*P*-value
Aortic cross-clamp time (minutes)	78.2 (9.1)	70.4 (8.5)	68.7 (7.9)	<.001
CPB time (minutes)	108.6 (11.2)	102.3 (10.5)	100.8 (9.8)	.004
Cardioplegia volume (mL)	320 (60)	500 (50)	400 (40)	<.001
Need for repeat cardioplegia dose, *n* (%)	47 (100%)	0 (0%)	4 (8%)	<.001
Need for defibrillation upon reperfusion, *n* (%)	9 (18%)	3 (6%)	2 (4%)	.034
Time to spontaneous sinus rhythm (minutes)	4.6 (2.2)	3.1 (1.6)	2.9 (1.7)	.002
Postoperative sixth hour troponin I (ng/mL)	6.8 (1.9)	4.2 (1.3)	4.0 (1.5)	<.001
Postoperative sixth hour CK-MB (U/L)	72.5 (15.6)	51.3 (12.1)	48.9 (10.4)	<.001
Lactate (postoperative sixth hour, mmol/L)	3.8 (1.0)	2.9 (0.8)	2.7 (0.7)	<.001
Maximum inotropic score (first 24 hours)	9.5 (3.1)	6.4 (2.2)	6.1 (2.0)	<.001
Duration of mechanical ventilation (hour)	16.8 (4.5)	13.2 (3.9)	12.9 (3.6)	.001
Reintubation after extubation, *n* (%)	5 (10%)	1 (2%)	1 (2%)	.09
Length of ICU stay (days)	4.2 (1.3)	3.5 (1.2)	3.4 (1.0)	.010
Hospital stay (days)	9.1 (2.5)	8.3 (2.3)	8.2 (2.0)	.071
Low cardiac output syndrome, *n* (%)	7 (14%)	2 (4%)	1 (2%)	.048
30-day mortality, *n* (%)	3 (6.4%)	1 (2.4%)	2 (4.5%)	.47
Delayed sternal closure	14 (29.8%)	9 (20.5%)	7 (16.7%)	.308

Data are presented as mean (SD) or *n* (%). *P*-values were derived from 1-way ANOVA (continuous variables) or chi-square/Fisher’s exact test (categorical variables).

**Figure 2.**
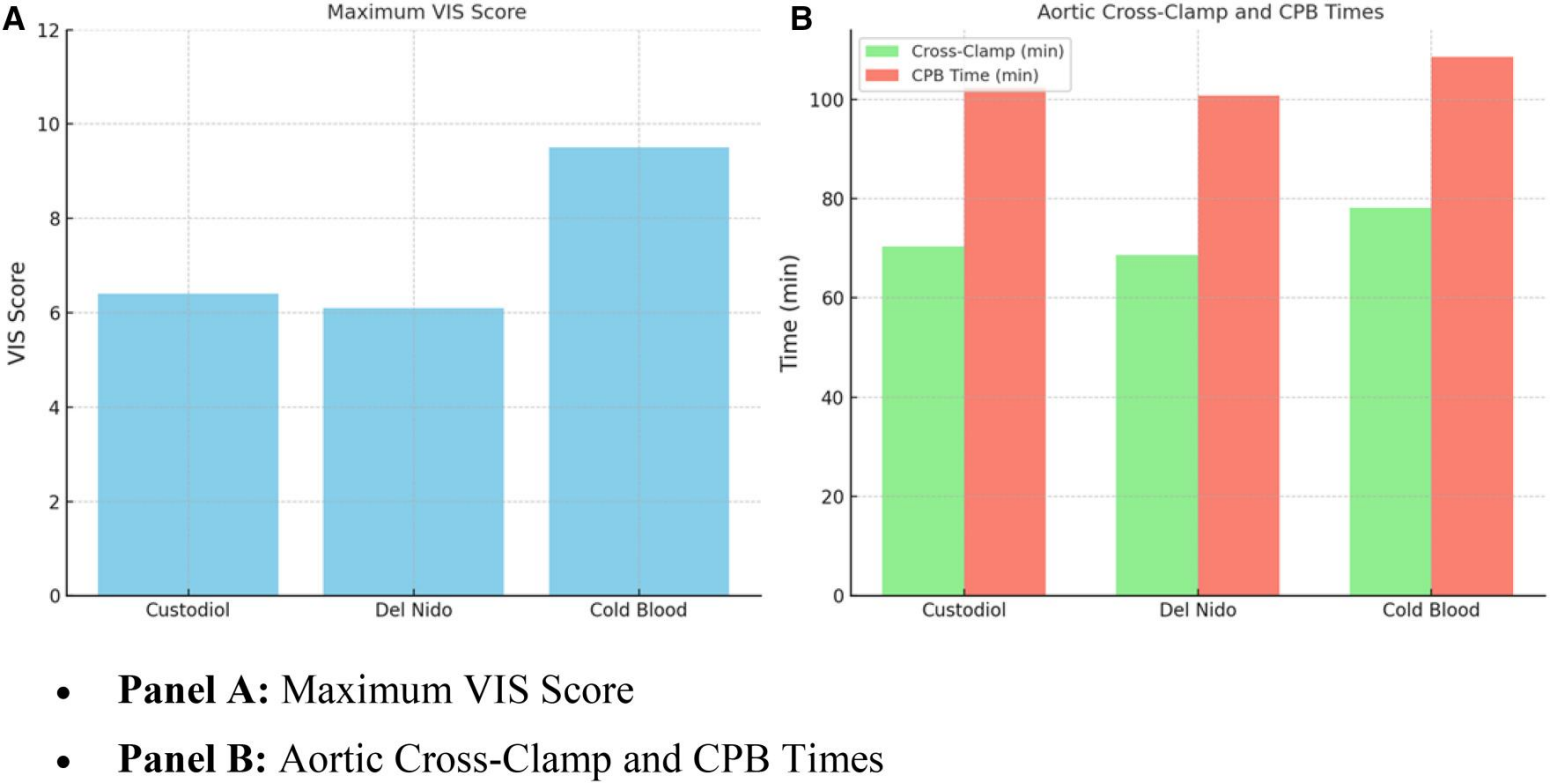
(**A**) Comparison of maximum vasoactive-inotropic scores (VIS) during the first 24 hours postoperatively among Custodiol, Del Nido, and cold blood cardioplegia groups. (B) Comparison of aortic cross-clamp and cardiopulmonary bypass (CPB) durations across cardioplegia strategies. Single-dose groups (Custodiol and Del Nido) showed shorter operative times. Error bars represent standard deviation

**Figure 3.**
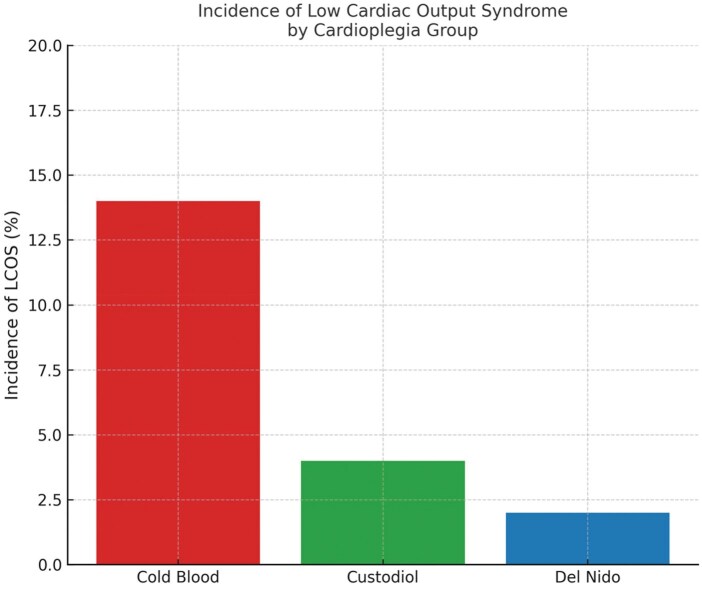
Incidence of Low Cardiac Output Syndrome (LCOS) Within the First 24 hours Postoperatively. Both Custodiol and Del Nido Groups Demonstrated Significantly Lower LCOS Rates Compared to the Cold Blood Cardioplegia Group (*P* = .048)

*Post hoc* comparisons for significant variables (**Table 4**) showed lower age, weight, and VIS scores in Custodiol and Del Nido versus cold blood (*P* < .05), with no differences between Custodiol and Del Nido. This supports their clinical comparability and reduced inotropic needs. Cardiopulmonary bypass and cross-clamp times differed significantly between cold blood and both the groups, but not between Custodiol and Del Nido. Early postoperative echocardiographic assessments (within 48 hours) showed preserved LVEF (≥55%) in 124 patients (93.2%), with no group differences (Custodiol: 94.4%, Del Nido: 95.5%, cold blood: 89.2%; *P* = .41). Mild pericardial effusion occurred in 9 patients (6.8%), needing no intervention. Transient ventricular dysfunction (EF <50%) affected 6 patients (4.5%), mostly in cold blood (*n* = 4, *P* = .26). Troponin I levels, measured at 6-72 hours (**Figure 1**), were lower in Custodiol and Del Nido than cold blood (*P* < .001). Cold blood had higher VIS (*P* < .001), indicating greater myocardial support need (**Figure 2A**). Custodiol and Del Nido had shorter operative times (*P* < .01), likely due to single-dose efficiency (**Figure 2B**). **Figure 3** highlights single-dose strategies’ myocardial protection.

**Table 4.** *Post Hoc* Pairwise Comparisons

Variable	Overall *P*-value	Custodiol vs Del Nido	Custodiol vs Cold blood	Del Nido vs Cold blood
Age	<.001	NS	*P* < .001	*P* = .002
Weight	.008	NS	*P* = .005	*P* = .011
VIS Score	.006	NS (*P* = .671)	*P* = .019	*P* = .004

*Post hoc* comparisons between the groups for significantly different variables are presented.

Abbreviation: NS = not significant.

### Troponin area under the curve and regression analysis

Troponin AUC, calculated over 72 hours, assessed myocardial injury. Mean AUC values were 14.2 (3.1) ng hour/mL (Del Nido), 14.7 (3.3) ng hour/mL (Custodiol), and 17.6 (3.9) ng hour/mL (cold blood) (*P* < .001). *Post hoc* analysis showed lower AUC in Del Nido and Custodiol versus cold blood, with no difference between them. Multivariable regression, adjusting for cross-clamp and CPB times (**Table S1**), confirmed Custodiol and Del Nido’s association with lower troponin AUC versus cold blood.

### Postoperative mortality analysis

Six neonates (4.5%) died within 30 days postoperatively. In the cold blood group, 3 deaths occurred: 2 after emergency surgery and ECMO for severe preoperative acidosis, on days 16 (sepsis, multiorgan dysfunction) and 25 (post-ECMO weaning); 1 on day 4 from ventricular fibrillation despite normal biventricular function. In the Del Nido group, 2 deaths occurred: 1 after ECMO for LCOS, on day 21 (multiorgan dysfunction); another with low birth weight (2.4 kg), on day 16 (sepsis, prolonged ventilation). In the Custodiol group, 1 death occurred after ECMO for severe preoperative acidosis, on day 23 (sepsis, multiorgan failure).

## DISCUSSION

Myocardial protection strategies in neonatal cardiac surgery remain heterogeneous, reflecting both the lack of consensus on optimal cardioplegia solutions and the unique challenges of the immature neonatal myocardium.^14–16^ In 133 neonates undergoing ASO, Custodiol and Del Nido cardioplegia outperformed cold blood cardioplegia, with shorter cross-clamp and CPB times, lower myocardial injury markers, and reduced inotropic needs, likely due to their single-dose design minimizing intraoperative interruptions.

Paediatric cardiac surgery preferences vary: North American centres favour Del Nido for simplicity and cost-effectiveness, European centres use both Custodiol and Del Nido,^15^ while Chinese hospitals prefer cold blood cardioplegia.^17^ Our cohort’s Custodiol and Del Nido groups had shorter operative times than blood (**Figure 2**).

Giordano *et al*^11^ reported similar Custodiol-related reductions but higher troponin I levels, unlike our findings. Floh *et al*^18^ found no operative time benefits with Del Nido, possibly due to case complexity. The neonatal myocardium’s vulnerabilities require tailored protection.^15–16^^,^^19^

Single-dose cardioplegia (Custodiol and Del Nido) reduces coronary manipulation, likely minimizing endothelial trauma in ASO coronary reimplantation. In our series, 19 patients (14.3%) with intramural LMCA and 16 (12%) with other coronary anomalies had no ischaemic events, suggesting single-dose strategies’ advantage in such cases.

Troponin I and CK-MB are key indicators of cardioplegia efficacy. **Figure 2** shows lower hs-cTnI levels at 6-72 hours in Custodiol (4.2 ± 1.3 ng/mL at 6 hours) and Del Nido (4.0 ± 1.5 ng/mL) versus cold blood (6.8 ± 1.9 ng/mL, *P* < .001), indicating better myocardial protection. Cumulative hs-cTnI AUC was lower in Del Nido and Custodiol than cold blood (*P* = .004), with no difference between them (*P* = .61).Single-dose cardioplegia’s favourable myocardial effects align with Pérez-Andreu *et al*^20^ (reduced enzyme leakage post-ASO with Custodiol) and Mohammed *et al*^13^ (lower troponin I with Del Nido). Yet, Bojan *et al*^16^ reported higher troponin I with Custodiol versus warm blood cardioplegia, indicating temperature and delivery protocols’ impact.

AUC analysis of troponin levels confirmed less myocardial injury in the Custodiol and Del Nido groups, supporting their clinical benefit. Mylonas *et al*^21^ found no troponin difference between blood and crystalloid cardioplegia, suggesting comparable modern techniques. Lower troponin likely indicates better myocardial preservation, evidenced by reduced inotropic needs and LCOS incidence, a key protective effect in high-risk neonates. Linear regression using troponin AUC as the dependent variable, with cardioplegia type, cross-clamp time, and CPB duration as covariates, confirmed Del Nido and Custodiol’s association with lower troponin release versus cold blood.

Single-dose cardioplegia enhances early postoperative recovery. The Custodiol and Del Nido groups showed faster sinus rhythm recovery and lower defibrillation rates, likely due to depolarized arrest (eg, lidocaine in Del Nido) reducing reperfusion arrhythmias.^6^^,^^13^

**Figure 3** shows lower maximum VIS in Custodiol (6.4 ± 2.2) and Del Nido (6.1 ± 2.0) versus cold blood (9.5 ± 3.1, *P* < .001), indicating less myocardial support need. This aligns with Mohammed *et al*^13^ and Pérez-Andreu *et al*,^20^ suggesting single-dose cardioplegia better preserves cardiac function. **Figure 3** shows lower LCOS incidence in Custodiol (4%) and Del Nido (2%) versus cold blood (14%, *P* = .048), highlighting single-dose strategies’ myocardial protection. Early postoperative echocardiographic parameters (LVEF, pericardial effusion, transient dysfunction) were similar across groups, supporting Custodiol and Del Nido’s protective effect. Dolcino *et al*^22^ noted prolonged cross-clamp times with Custodiol may increase LCOS risk, not seen in our cohort due to strict ischaemic time control. Floh *et al*^18^ found no decrease in ventricular dysfunction with Del Nido, highlighting the need for standardized outcome measures. No 30-day mortality differences (*P* = .47) across the groups confirm the safety of all 3 strategies. Two emergency surgery/ECMO patients, included to reflect real-world practice, may affect early outcomes but were noted as a limitation. Their inclusion enhances external validity, though future studies may exclude such high-risk cases to reduce confounding.

Each cardioplegia strategy has unique strengths and weaknesses. Custodiol’s single-dose protection (up to 120 minutes) simplifies complex coronary transfers.^19^ Its high volume may cause hemodilution, risking postoperative anaemia, oedema, or hyponatremia, potentially leading to seizures.^7^^,^^19^^,^^23^

Dolcino *et al*^22^ noted prolonged cross-clamp times with Custodiol may increase neonatal LCOS risk, requiring careful ischaemic time monitoring. Münch *et al*^24^ emphasized Custodiol’s efficacy relies on strict 4-8 °C temperature control to prevent rewarming-induced myocardial arrest failure and reperfusion injury. MUF and precise temperature management mitigate these risks.^4^^,^^5^^,^^25^ Del Nido’s blood-crystalloid mix reduces hemodilution, offering 60-90 minutes of protection,^5^ with lidocaine-induced hyperpolarized arrest likely minimizing reperfusion injury, supported by our lower troponin levels.^12^^,^^13^

Cold intermittent blood cardioplegia, though needing frequent dosing, benefits from oxygenated perfusate and familiarity, suiting prolonged procedures.^14^ Its repeated coronary manipulations risk endothelial trauma in neonates with small coronary ostia. Our 4.5% mortality rate matches ASO standards,^11^^,^^13^ with no coronary-related deaths. Anatomical risk factors (eg, single ostium, intramural coronaries, arch anomalies) were evenly distributed across groups (**Table 3**), ensuring cohort comparability. This supports the validity of differences in myocardial injury markers and early outcomes. Baseline differences (age, weight) limit findings, warranting multivariate analyses in future studies.

*Post hoc* analysis revealed cold blood patients were older, heavier, with higher VIS, while the Custodiol and Del Nido groups were similar. This indicates their clinical comparability, suggesting outcome differences are not solely due to baseline variability. An ongoing trial^26^ could identify optimal strategies.

Custodiol and Del Nido improve ASO efficiency and outcomes. Surgeons should tailor cardioplegia to patient factors with strict protocols for optimal myocardial protection. Our observational design, lacking multivariable adjustment, limits causal inference. Though ventilation duration and ICU stay differences were significant, their individual clinical impact is modest. In neonatal care, small ICU resource savings can yield significant benefits in high-volume centres or fragile populations, balancing our interpretation.

## CONCLUSION

Neonatal myocardial protection remains a critical challenge in ASO due to the immature myocardium’s susceptibility to ischaemic injury. This retrospective multicentre study of 133 neonates suggests that Custodiol and Del Nido cardioplegia may offer improved efficiency and outcomes, including shorter cross-clamp times, less myocardial injury, and reduced inotropic needs. Each strategy—Custodiol’s prolonged single-dose protection, Del Nido’s balanced blood-crystalloid composition, and cold blood’s oxygenated perfusate—offers unique advantages, but also limitations such as hemodilution, reperfusion injury risk, or frequent dosing needs. Surgeons should consider tailoring cardioplegia selection to patient-specific factors, including coronary anatomy and anticipated surgical complexity, while ensuring meticulous delivery to optimize myocardial preservation. Given the observational nature of this study, these findings should be interpreted with caution and confirmed in prospective randomized trials.

### Strengths and limitations

This study’s strengths include its multicentre design, standardized operative protocols, and comprehensive postoperative biomarker analysis. However, the retrospective nature of the analysis, small sample size, and lack of multivariable adjustment limit the strength of causal inference. Given the inherent limitations of observational design and absence of multivariable adjustment, the results should be interpreted as associative rather than definitive.

## Supplementary Material

ivaf215_Supplementary_Data

## Data Availability

The data underlying this article are available in the article and its supplementary materials. Further details can be provided by the corresponding author upon reasonable request.
