# Supplementary material for: Myocardial Protection Efficacy of Custodiol, Del Nido, and Cold Intermittent Blood Cardioplegia in Arterial Switch Operation
Source: Interdiscip Cardiovasc Thorac Surg. 2025 Sep 24;40(12):ivaf215. doi: 10.1093/icvts/ivaf215 (PMC12782733; doi:10.1093/icvts/ivaf215)
Supplement: ivaf215_Supplementary_Data [file ivaf215_supplementary_data.zip › Supplemantary Table S1.docx]

Supplemantary Table S1. Multivariable Linear Regression Analysis for Troponin AUC

| **Predictor Variable** | **β Coefficient** | **Standard Error** | **95% CI** | **p-value** |
| --- | --- | --- | --- | --- |
| Del Nido vs. Cold Blood | –1.35 | 0.45 | –2.22 to –0.48 | 0.002 |
| Custodiol vs. Cold Blood | –1.18 | 0.50 | –2.17 to –0.19 | 0.021 |
| Aortic Cross-Clamp Time (min) | +0.09 | 0.03 | +0.03 to +0.15 | 0.004 |
| CPB Duration (min) | +0.07 | 0.02 | +0.03 to +0.11 | 0.001 |
| Intercept | 6.52 | 0.71 | 5.12 to 7.91 | <0.001 |
